# Can Air Quality Citizen-Sensors Turn into Clean Air Ambassadors? Insights from a Qualitative Study

**DOI:** 10.3390/ijerph181910046

**Published:** 2021-09-24

**Authors:** Guilhem Dardier, Françoise Jabot, Flora Pouliquen

**Affiliations:** 1 EHESP, F-35000 Rennes, France; francoise.jabot@ehesp.fr (F.J.); florapouliquen@orange.fr (F.P.); 2 Univ Rennes, EHESP, CNRS, ARENES—UMR 6051, F-35000 Rennes, France

**Keywords:** citizen sensing, clean air ambassador, air quality, peer education, peer mediation

## Abstract

While the figure of ambassador is being increasingly called upon in the field of environmental health, its scope remains fuzzy and its success factors have been little studied. This article presents the results of a qualitative study performed over three years on a French citizen-sensor scheme for air quality. The scheme draws on volunteer citizens to measure fine particles by means of micro-sensors. Volunteers are also tasked with raising awareness in their entourage about environmental issues with the aim of changing people’s behaviour. We investigated this strategy and sought to identify the conditions that enable citizens to become effective clean air ambassadors. The scheme’s intervention logic was first reconstructed and a literature review of similar projects was conducted. Then, three surveys were carried out with the scheme’s volunteers. Each survey consisted of an observation of the volunteers’ practices and individual interviews (70 in all) in order to understand these citizen-sensors motivations and experiences, and characterize how they fulfilled their role. We concluded that, for citizen sensing, the scope and role of ambassador should be reconsidered insofar as these citizens serve as peer leaders and mediators. In this respect, we try to define the success factors for citizen-sensing-based ambassadors programmes.

## 1. Introduction

Air pollution is one of the main causes of excess mortality and loss of life expectancy worldwide [1]. Every year, it causes between 4.2 and 8.8 million premature deaths throughout the world, most of which are related to cardiovascular [2,3] and respiratory diseases [4,5]. In France, air pollution is responsible for more than 48,000 deaths per year, half of which occur in conurbations with more than 100,000 inhabitants [6]. In one such conurbation, regularly affected by episodes of air pollution by nitrogen dioxide (NO_2_) and fine particles (PM2.5 and PM10), the City Council has made improving air quality one of its priorities, in line with those of the French Healthy Cities network. To this end, in 2016, the City Council set up a pilot scheme to change the behaviour of inhabitants in favour of air quality. The project draws on citizen volunteers who measure air quality using fine-particle micro-sensors (AirBeam1 and 2), which is conducive to raising people’s awareness about related issues. These volunteers will, in turn, be able to raise awareness among those they know, acting as clean air ambassadors. 

The concept of ambassador, historically used in marketing or by charities, has gained momentum in the fields of health promotion [7,8] and the environment [9,10]. Much research has focused on assessing the effects and limitations of ambassador-based programmes in these two areas [9,11,12,13], but it does not address the factors behind the effectiveness of such programmes. While the ambassador’s roles are clearly identified (i.e., committing to an environmental issue, sharing experience, educating and encouraging others to adopt pro-environmental behaviour), very little empirical research has been conducted into the figure of the environmental health ambassador in relation to his or her different roles. It appears all the more crucial to address this knowledge gap as it intersects with the current limitations of the literature on community-based monitoring projects. As these projects have developed throughout the world, there has been increasingly more work undertaken to identify them [14,15], to assess the technical performance of their micro-sensors [16,17,18,19] and to characterise their individual, social and political effects [20,21,22]. At the individual level, engagement in such projects has been related to behavioural and attitude changes, as well as gains in health literacy, social capital and efficiency of civic participation [21,22,23]. At a social level, such projects have been described to enhance the public knowledge of air pollution and raise awareness about related issues [19,22,24,25]. At a political level, they can lead to a change in environmental policies and local risk governance through mechanisms such as collective empowerment, community building and improvement in air quality data [20,21,26]. However, implementation and the situations that induce success in such projects are mostly overlooked, as are the social dynamics at play in citizen-sensor communities [24,27,28]. The literature also points out both the ethical and technical issues faced by citizen sensing projects [16,17,24,29]: data quality, reliability and reproducibility; equal access for all citizens to these innovative technologies; data protection and respect of privacy, especially when real-time, geolocalised, exploitable big data are produced. It also appears that their effects on individual behaviours strongly depend on their implementation, with a risk of watering down their positive outcomes or even generating negative ones. Because it is a complex process [30], behavioural change may not be triggered by the simple use of a micro-sensor [23,24], and when it does, mechanisms are still poorly understood [31]. If not properly explained, the raw information about air pollution produced by the micro-sensor can even generate anxiety and unethical behaviour [32], which highlights the importance to train and support citizen sensors. In the same way, the social and political implications of citizen sensing projects seem to depend on their goal and design, and more specifically on how citizens are considered and involved. In this respect, it is possible to identify four different types of projects that rely on citizens and micro-sensors, and divide them into two categories. On the one hand, environmental monitoring initiatives and citizen science projects are mostly community-based and -led, sometimes with the support from NGOs or members of academia. They are mainly focused on the process to build citizen expertise and empowerment, through data collection, knowledge garnering, skills acquisition and community building [33,34,35]. Safecast in Japan [36], the PetaJakarta.org project in Indonesia [37] and the Airplane Monitor Schiphol in the Netherlands [20] are recent examples of community-based environmental monitoring, while Citoyens Capteurs in France, Smart Citizen and Making Sense in Europe [38] or Citizen Sense in England and the USA [39,40] are all examples of citizen science projects using micro-sensors. On the other hand, institutional and academic citizen sensing projects, generally initiated by municipalities or researchers, primarily aim at testing micro-sensors and at using their potential to change behaviours and raise awareness about air quality [24,41]. Some examples of those types of projects are CitiSense in San Diego [42], the Array of Things in Chicago [29], or Mobicitair in Grenoble. Therefore, to question the success of any project involving citizens and micro-sensors, it appears crucial to first study its rationale, the role devoted to citizens and the strategies implemented to foster their individual and collective engagement.

The French citizen sensing project draws on an intervention research project [43]. From its beginning, a team of researchers has been mobilised to produce knowledge on the rollout of the experiment and to support decision-makers in continuing and adjusting the project. Drawing on the research conducted, this article seeks to investigate the project’s strategy: to what extent and under what conditions do citizen volunteers become clean air ambassadors? After explaining the project’s logic, we will present the results of three surveys carried out among its volunteers and then discuss the conditions for the effectiveness of its strategy.

## 2. Materials and Methods

Firstly, work was carried out to reconstruct the intervention logic of the project in the form of a logic model [44]. This model describes the chain of effects caused by the implementation of measures or action. It draws on the analysis of project documents and interviews with those responsible for the local air quality policy. They validated the model. 

Secondly, a review of the literature dealing with citizen-sensor networks for air quality was carried out in order to identify similar projects and to draw lessons from the strategies developed to encourage citizen participation. Bibliographic searches were executed in the main French and international academic databases (Cairn, Google Scholar, PubMed, and Scopus), with the following keywords: air quality, air pollution, micro-sensor, low-cost sensor, citizen sensing, monitoring, citizen science, community-based, participation, empowerment, effects, effectiveness, implementation, evaluation. These keywords were used in French and English, both singular and plural. They were associated in different combinations, using Boolean operators “AND” and “OR”. Relevant articles were selected based on their titles and abstracts. A second review was later conducted, focusing on the concept of environmental health peer mediators. The aim was to clarify the concept and identify the conditions for such mediation. The same research protocol was applied, with only the keywords changing: mediator, peer, ambassador, health, environment, air quality, education, mediation, promotion, evaluation, effectiveness, implementation. 

Thirdly, three surveys were conducted on the volunteers of the project in April 2017 (volunteers in 2016–2017), April 2018 (volunteers in 2017–2018) and April 2019 (volunteers in 2018–2019). They were designed to better understand and characterise how they carried out their missions. These surveys were based on the observation of collective activities with the volunteers, combined with individual semi-structured interviews with these volunteers. The interviews’ time and place were left at the volunteers’ convenience. They lasted between 40 min and two hours, and were recorded with the consent of the individuals, transcribed, anonymised and coded. The themes explored during the interviews were as follows: motivation to join the project, use of the micro-sensor, involvement in project activities, view of and investment in the role of clean air ambassador, the impact of the project on the volunteer and his or her entourage. For each of the three years, a thematic analysis of the interviews was carried out, based on the cross reading of the notes taken during the exchanges and of the interviews transcriptions and their thematic encoding. The results of this year-by-year analysis were then compiled in a table, which made it possible to perform an overall analysis of all three years as well as a comparison between them. The observation of collective activities was used to collect complementary data regarding the volunteers’ involvement in project activities: organisation and animation modalities of the events, audience and topics covered, institutional and social dynamics at play. These data were compiled and analysed by year and by type of activity (training session, collective measurement, discussion meeting). 

## 3. Results

### 3.1. The Intervention Logic of the Project and Its Place in Local Policy

**Figure 1:**
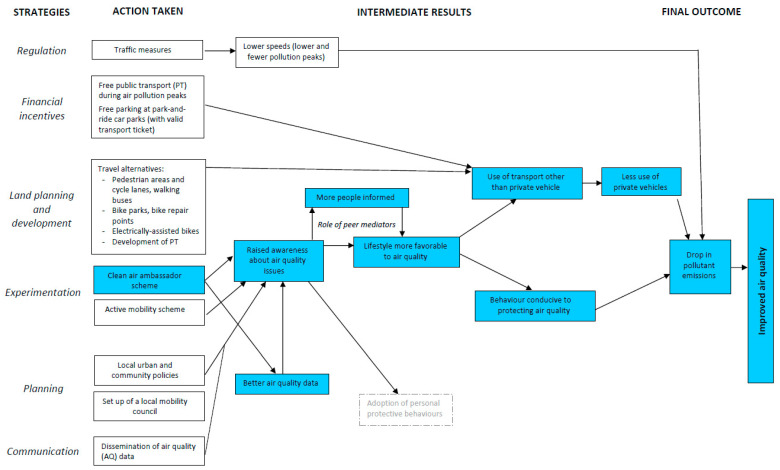
The intervention logic of the project and its place in local policy.

Local air quality improvement policy includes different categories of measures: regulation, financial incentives, land use and development, experimentation, planning and communication. These measures aim to reduce the speed of traffic, foster the use of alternative modes of transport (with less use of private motor vehicles) and raise awareness of air quality issues. The combined effect of these three outcomes should lead to a reduction in local emissions of atmospheric pollutants, and therefore to an improvement in local air quality (Figure 1).

The citizen sensing project is a component of this policy. It aims at changing individual behaviour, particularly in terms of mobility and heating. These changes in behaviour first require individuals to be aware of the issues relating to air pollution. The clean air ambassadors became aware of these issues by participating in the scheme, and the general public could be informed via the city council communication and by the ambassadors. The latter are therefore a key component of the project as their role is to help increase local air quality data and to raise awareness in their entourage. 

### 3.2. The Profile and Motivation of the Volunteers

Of the 101 volunteers who took part in the three phases of the experimentation, 70 were interviewed individually. Of these, 39 were men and 31 were women, aged between 28 and 73 years. They mainly had intermediate and higher socioprofessional categories. 48 had a professional activity, 15 were retired, one was a student and six did not specify their status (Table 1).

All of them joined the project because they had an interest in the environment, which often goes hand in hand with ecological behaviours (regular use of a bicycle, ethical consumerism, involvement in environmental or health action: promotion cycling, defence of natural areas, raising public awareness of respiratory diseases, etc.).

A few volunteers had expertise in technical fields related to the project: eight worked in the digital sector and were particularly interested in measurement methods and data processing; one was involved in the project’s technical development and had developed his own sensor.

The volunteers expressed various reasons for participating in the project. The first reason, expressed by 87% of them, was to evaluate their own exposure to fine particles, to “find the truth”. This may be fuelled by collective representations relating to the nearby ring road, the presence of a busy park-and-ride car park and a controversy over the emissions from a local incinerator. Exposure was also of interest to those who practised a physical activity in the city, such as cycling, as they wished to evaluate risk and identify the safest areas for such activities. Volunteers with children were particularly concerned about the risk of their children being exposed to air pollution. Lastly, some ambassadors saw the sensor as a good means to better understand the link between the onset of respiratory symptoms and the level of pollution.

In addition, 50% of the volunteers considered that their involvement in the project was part of a collaborative bottom-up approach—one in which they would contribute directly to the measurements, particularly in areas not covered by the official measures, and in which they would work to change people’s behaviour (Table 2). “Sometimes you get surprising things (…) that’s why I try to make it [the sensor] work as well as possible. If you don’t record these things, they’ll go unnoticed”. The proportion of volunteers who declared a collective motivation went from 75% in the first year to 33% in the third year.

### 3.3. Volunteer Activities

#### 3.3.1. The Use of the Sensor for Measuring Air Quality

Ninety percent of the ambassadors used their sensors to carry out mobile measurements outdoors, during their usual daily journeys (commuting, the school run, walking, etc.). Several volunteers carried out additional measurements at night or by extending their usual route to collect more data. However, most volunteers lost motivation during the project, with a drop in measurement activity after one or two weeks.

Sixty-five percent of the ambassadors also carried out fine particle measurements inside their homes and compared them with outside measurements (Table 2). It should be noted, however, that this percentage fell from 100% of the volunteers for the first year to 40% for the second year and 67% for the third year. This drop in the indoor use of the fine particle micro-sensor may have been due to the volunteers in the second year and beyond being able to borrow an additional sensor that measures the concentration of CO_2_ in a room.

#### 3.3.2. Participation in Collective Activities

In the first year, the volunteers were given several compulsory training sessions organised by the approved organisation for monitoring air quality in the region. The aim of these sessions was twofold: to brief volunteers about air quality and to teach them how to use the sensors. Volunteers were also given the opportunity to discuss the data collected. Collective measurements were organised for a specific time of day that the group of volunteers chose together. The same activities were repeated in the second year, with an optional midterm discussion meeting as part of the volunteer support system, but most of them were dropped for the third year or became optional. As a result, only 27% of the third year volunteers stated that they had taken part in at least one of the activities (vs. 100% for the first two years) and 38% of them said that the project had given them a better understanding of air quality (vs. 54% for the first two years). All years alike, some participants thought that the scheme enabled them to better identify relevant information sources, understand and sort information and to feel more legitimate to talk about and act upon air quality.

### 3.4. The Perception of the Role of Clean Air Ambassador

#### 3.4.1. A Sense of Individual Responsibility

The volunteers understood the role of clean air ambassador to be one that carries out measurements, which can respond to an individual or collective purpose. This is so regardless of whether one is measuring to set an example, to serve one’s community or to increase the amount of data for a particular area. In the first year, several volunteers saw themselves as “pioneers” with several responsibilities: to make the project known beyond the city, to act in line with their moral values, to carry out measurements regularly in order to produce a large quantity of data to be exploited. “I take on a responsibility; they give me a sensor and expect me to provide actionable information, and then to raise awareness among other people”. Sometimes, when volunteers encountered a technical problem with the sensor, they were discouraged and even felt they had “let the side down” by not having been able to fulfil their role as citizen-sensors. This feeling of responsibility led some of them to change their behaviour with regard to the information collected: 24% of the volunteers reported having adopted individual outdoor protection measures (changing their cycling routes and times, changing how they breathe during exercise, etc.). This number more than doubles went it comes to indoor air quality: 53% admitted to having changed their ventilation practices or reduced the use of scented candles and incense. According to the volunteers, three factors seem to be at play to explain this difference between indoor and outdoor air quality behavioural responses: the difficulty to connect outdoor air pollution to specific settings and behaviours, the incapacity to feel individually responsible since they “do a lot already”, and the perception to be powerless at an individual level. Moreover, those behaviour changes concerned a very limited portion of the local population, with a lack of data concerning the behaviours of the rest of the population during the same time span. Therefore, it is not possible to identify the changes in road traffic or fine particles local concentrations that could be attributed to the scheme [45].

#### 3.4.2. Difficulties in Taking on the Role of Raising Awareness in Their Entourage

In addition to measuring air quality, the volunteers think of the clean air ambassador as someone who can raise the alarm, someone who actively raises awareness in his or her community. They described this as “giving impetus to improving air quality”. The volunteers less invested this second aspect of the ambassador’s role, especially those of the second and third years of the project. Thus, 54% of them stated that they had not properly fulfilled this duty of awareness-raising and passing on information (Table 2). To justify this, they put forward three main arguments. Firstly, they struggled to use the sensor when addressing their peers (should I let the sensor “speak” for itself, or should I use it to support my case? Should I pass on an external message or create my own story?). Secondly, they thought it was complex to develop a technically well-founded, convincing but not guilt-tripping argument. Lastly, the faced logistic issues relating to the technical system and its many malfunctions.

Some volunteers describe a two-stage involvement. Firstly, they made an effort to communicate with their relatives: “The children participated, they saw the box, they understood the explanations and we took the measurements together”. Next, they talked about their measurements and distributed project posters in their social sphere, at work, in the neighbourhood, with parents of school children and within associations. They reported that people were generally interested and wanted to know the current level of pollution and the differences between areas. One volunteer said that he had become the “pollution officer” in his neighbourhood.

The influence of the ambassadors on their entourage, via their role of standard-bearers, is difficult to appreciate through their messages and arguments. Only five volunteers believe that their participation in the project will have a real influence on their entourage, considering that behaviour change is more of a process than a breakthrough.

## 4. Discussion

### 4.1. The Project’s Strategy in Question

#### 4.1.1. The Ambassador’s Position, between Peer and Mediator

These findings raise a number of questions about the project’s strategy, namely to encourage people to adopt behaviour that is favourable to air quality with the help of volunteers. As previously mentioned, the ambassadors are both the target of the intervention and the intermediary with their entourage. The use of sensors increases their awareness and enables them to convey messages to their community that are more likely to convince than if they came from someone else, such as from experts or politicians [46,47]. Where one neighbourhood resident raises another resident’s awareness, this can make environmentally friendly behaviour come across as an acceptable social norm, as shown by the experience of Hopper and Nielsen [48] on waste recycling [49]. Thus, the ambassadors’ action in their community is based on a twofold strategy: peer education and mediation between the sensor to be used, the messages to be conveyed and the people to inform. If the volunteers are to fulfil their role as peers and mediators, then, as shown by the literature [10,46,47,50], several conditions must be met in terms of recruitment, training and support, and the implementation of mechanisms that are essential to building their individual and collective identity. The question here is to see whether the project creates these conditions.

#### 4.1.2. A Targeted Recruitment Process

Volunteers must be recruited according to their profile and their motivation to join the project and operate in targeted environments (school, work). This ensures that they believe in the effectiveness of the activities and that they share certain sociodemographic characteristics (gender, age, place of work or study) with each other and with the intended beneficiaries of the action [46,50]. It is also a question of recruiting individuals with social and communication skills, or even a socioprofessional position that strengthens their ability to lead change [10]. In the project, all the volunteers were accepted, provided they lived in the metropolitan area. This lack of selection may have led to two drawbacks. Firstly, the volunteers were possibly too distant with one another, and with the intended beneficiaries of the action. Secondly, some volunteers did not wish to work on raising awareness in their entourage: very few people justified their involvement in the project by a desire to bring about a change in people’s behaviour. Rather, the volunteers mainly expressed personal considerations to justify their participation in the project, which is in line with the conclusions of recent studies on volunteers in projects using sensors [21,28].

#### 4.1.3. Tools and Adequate Training

Volunteers must be properly equipped for awareness raising [51,52]. The micro-sensors given to them can be effective in raising awareness in their entourage as such devices arouse people’s curiosity through their fun aspects. They also serve to reduce the psychological distance [53] between their users and air pollution, by anchoring the experience of pollution in the users’ daily lives and linking them to familiar places. This allows volunteers to base their arguments on precise, local and concrete data (*legitimacy of argument*), to have a personal commitment to share—a self-narrative—and thus to feel more at ease in seeking to convince (*legitimacy of position*). However, this tool must come with training so that volunteers have the skills needed to mediate between the data produced by their sensor and people in the community with very disparate levels of knowledge and interest in air quality [46,51]. Although volunteers received training at the start of the project, this was limited to the technical aspects of the measuring and did not cover the social dynamics involved. As a result, several volunteers felt that they had no real legitimacy and had difficulty finding the “right position” and arguments with regard to their entourage.

#### 4.1.4. Quality Information and Involvement in the Knowledge Production Process

Moreover, volunteers must have good information on air quality and be involved in the entire data and knowledge production process, from the expression of needs to the dissemination of results [33,35]. Most air quality citizen-sensor schemes generally aim to make air quality information accessible to the public. The work by Morawska et al. [19], which analysed 17 projects using low-cost sensors, concludes that these technologies have the potential to “expand the conversations with communities”. The work by Mahajan et al. [25] stresses the possibility they offer of “enhancing public understanding of air pollution” when they are mobilised as part of a citizen science approach. In the studied scheme, although the results of the surveys confirm that most of the volunteers now have a better understanding of air quality (*knowledge acquisition*) and that some of them are more able to obtain and sort out information by themselves (*health literacy*), they do not necessarily suggest that information on air quality is circulating on a wider scale. This limitation of the project is undoubtedly because it is not part of a bottom-up citizen science approach but rather a top-down institutional citizen sensing approach [54].

#### 4.1.5. Leverage for Building an Individual and Collective Identity

The literature highlights the essential nature of peer recognition among themselves and by the general public [46,51]. This recognition is necessary for building individual and collective identity, and thus for creating meaning within a community and in its dialogue with the outside world [55]. Visual identification elements, formal gatherings and a participatory geographic information system (GIS) can provide effective support for establishing citizen-sensor networks [20] by sharing data, feedback and experiences. They can also foster the use of the micro-sensor in itself since social ties play a role in user acceptance of new technology [56], even offsetting the technical characteristics (and, potentially, the technical issues) of the technology to explain a positive attitude towards it, and therefore its positive deployment. In the first year, a feeling of responsibility, pride and belonging to a community initiative came about for various reasons: the limited number of volunteers, the innovative nature of the approach, the existence of collective training time and measuring and the direct posting by several volunteers on social media and their visibility in the local media. In contrast, during the second and third years, the same feeling of belonging was hampered by the technical limits of the scheme’s participatory GIS, the lack of common monitoring places and the lack of visibility perceived by the volunteers.

#### 4.1.6. Complementarity with Other Action

The peer mediator action should complement other strategies and achievements aimed at changing the environment in which the peers operate. A number of studies have called for greater clarity about the motivations behind peer education programmes for civic participation [57,58]. Is it the desire to democratise and improve public policies or to increase their social acceptability while reducing their cost? With regard to the project, local policy makers like to point out the scheme’s complementarity with the other components of the local air quality improvement policy and the experiments in urban planning and development (Figure 1). However, there is no formal link. The governance bodies are separate and the data collected by the volunteers are not used as an input for local air quality surveillance and management, nor to fuel any urban development project or environmental policy.

Overall, peer mediation depends on a number of conditions, not all of which were met here. This limited the feeling of self-efficacy in many volunteers [59] and hindered their commitment to awareness raising in their entourage. Consequently, the project was forced to review its objectives and adapt its strategy.

### 4.2. Limitations of the Survey

The scope of this study is limited in that the group did not reflect the sociodemographic characteristics of the general population, nor this population’s pre-existing awareness of environmental issues related to air quality. This low representativeness, which makes it difficult to extrapolate the results observed within the volunteer sample, was noted in the CitiSense mobile air quality sensing project in San Diego [42].

Nor is this group representative of the population of the area covered by the project territories, which, for the first two years, were underprivileged neighbourhoods. The volunteers’ views do not therefore reflect those of the residents living in these neighbourhoods. While low socioeconomic status communities are more vulnerable to air pollution [60] due to a range of factors (health status, lifestyle, access to healthcare, housing quality, working conditions), they perceive environmental issues from a greater distance and show a greater lack of control over these issues [61].

Lastly, as the study sample included people who had been involved in the project design, a complacency bias cannot be ruled out.

## 5. Conclusions

The citizen-sensor framework broadens the role of the ambassador. Indeed, his or her role is no longer limited to making the general public aware of a predefined issue through exemplary conduct and personal account. Now, it also includes sociotechnical mediation work between a sensor and the people it interacts with. Under this framework, the ambassador takes on the role of peer mediator, whose effectiveness is based on several conditions that we tried to define here, inspired, in particular, by peer health education. Such lessons can be useful for any community wishing to set up a citizen-sensing-based ambassador programme to raise public awareness and empower citizens about an environmental health issue (air, noise, odours, seismic risks, etc.). They are also useful for researchers wishing to study the conditions of implementation and the factors for the effectiveness of such interventions.

Moreover, in line with recent work on the subject [20,21,24], this study questions the social and political implications of citizen-sensor networks, and more specifically the concept of citizen science and its place in environmental policies. By investigating the links between technology, citizenship and policy making, citizen sensing is ultimately part of the current conversation on the smart city and its possible models. Is citizen sensing meant to serve a sensible city that seeks to use micro-sensors to better understand how the city functions, how to optimise it and offer urban services [62]? Or is it meant to serve a smart enough city that aims to use micro-sensors to empower citizens and enable them to influence public policy [29]? The answer to this question would inform us as much about the political status of citizen sensing projects, which is still unclear, as about the real implications of these different smart city models for the local governance of environmental risks.

## Figures and Tables

**Table 1:** Clean air ambassadors’ sociodemographic information.

Sociodemographic Characteristics	Project’s Volunteers		Metropolitan Area	France
	*n*	%	%	%
**Gender identity**				
Female	31	44	**52.6**	**51.6**
Male	39	**56**	47.4	48.4
Non-binary	0	0	/	/
**Age**				
Under 15	0	0	14.5	18.1
15–29	2	3	**33.2**	17.6
30–44	26	**37**	18.6	18.8
45–59	17	24	15	**19.9**
60–74	16	23	11.2	16.2
Over 75	0	0	7.5	9.3
Non-specified	9	13	/	/
**Socioprofessional category**				
Higher	21	**30**	16.8	13.1
Intermediate	19	27	14.2	14.7
Lower	8	11	22.9	27.1
Retired	15	21	19.1	**32.5**
Others (including students)	1	2	**27**	12.6
Non-specified	6	9	/	/

**Table 2:** Clean air ambassadors’ key interview responses.

Clean Air Ambassadors’ Interviews Main Results (Number of Respondents = 70)
**Motivations**
**87%** of volunteers explained their involvement in the project with personal considerations (to quantify their own exposure; to better understand the onset of breathing problems; to make a case against a specific infrastructure like a ring road or an incinerator)
**50%** of volunteers explained their involvement in the project with collective considerations (to be part of a collaborative, citizen-oriented project; to cover areas with poor air quality data; to contribute to better informed political decisions)
**Activities**
**90%** of volunteers carried out mobile fine particle measurements outdoors
**65%** of volunteers carried out fine particle measurements inside their home
**Outcomes**
**46%** of volunteers said that the project had given them a better understanding of air quality and tools to better find and sort out related information
**24%** of volunteers reported that they had adopted individual outdoor protection measures (changes in breathing practices; new times and routes for physical activities)
**46%** of volunteers stated they had fulfilled their duty as a clean air ambassador (awareness-raising and passing of information)
**7%** of volunteers considered their participation in the project will have a real and lasting influence on their entourage

## Data Availability

The data presented in this study are available on request from the corresponding author. The data are not publicly available due to personal data protection.
